# Performance of posterior 3D printed resin-matrix ceramic crowns fabricated in a fully digital workflow: a two-year prospective clinical study

**DOI:** 10.1007/s00784-026-06883-y

**Published:** 2026-04-21

**Authors:** Marina del Piñal Pellón, Celia Tobar, Pedro Díaz, Jesus Pelaez, Maria J. Suarez

**Affiliations:** Department of Conservative Dentistry and Prosthodontics, Faculty of Odontology, University Complutense of Madrid, Madrid, Spain

**Keywords:** 3D-printing, Resin-matrix ceramic, Crowns, Survival, Clinical evaluation

## Abstract

**Objectives:**

To evaluate the survival and success rates, as well as mechanical and biological outcomes of posterior 3D-printed resin-matrix ceramic crowns in a fully digital workflow over a 2-year follow-up.

**Material and methods:**

A prospective clinical trial was conducting involving 30 posterior crowns fabricated from a resin-matrix ceramic using DLP 3D-printing technology. Dental preparations were performed and scanned with an intraoral scanner by a single operator. All crowns were cemented using the same dual-curing resin cement. Clinical performance was assessed using California Dental Association (CDA) criteria. Periodontal parameters (plaque index, gingival index and probing depth) were evaluated with a periodontal probe at cementation and at 6-month, 1-year, and 2-year recall appointments on abutment teeth and contralateral or antagonistic uncrowned natural teeth used as controls. Data were analyzed using Wilcoxon signed rank test and Kaplan–Meier survival analysis.

**Results:**

The 2-year survival rate was 93%, and the success rate was 87%. Two crowns debonded, and no biological complications were observed. All crowns remained within the satisfactory range after 2 years. A slight yellow shift was detected in 4 crowns, resulting in a significant color change at 2 years (*p* = 0.046), while all other CDA parameters remained unchanged. The margin remained stable throughout the observation period. Plaque index increased after one year in the abutment and control teeth.

**Conclusions:**

Within the limitations of this study, including the absence of a control group, 3D-printed resin-matrix ceramic crowns may represent a viable alternative for posterior teeth. Long-term studies are required to confirm these results.

**Clinical relevance:**

Posterior 3D-printed resin–matrix ceramic crowns within a fully digital workflow demonstrated satisfactory performance after two years, supporting their potential as a viable option for posterior restorations.

## Introduction

In recent years, the increasing demand for highly esthetic restorations and the rapid development of digital technologies has driven significant progress in the research and development of novel ceramic materials. These new materials aim to enhance biocompatibility, achieve a natural tooth-like appearance, enable reliable bonding with composite agents, and overcome the inherent brittleness that has long limited their universal application [1–4].

Ceramic restorations offer several advantages over conventional metal-ceramic crowns, including being less invasive and presenting a lower risk of complications such as the detachment of the veneering porcelain by chipping [5, 6]. Zirconia, in particular, has shown excellent mechanical properties [7–10]. More recently, resin-matrix ceramics have been developed in an attempt to obtain a material that simulates dentin´s modulus of elasticity while remaining easily repairable. These materials are characterized by high dimensional stability and flexural strength, easy handling, and predictable outcomes due to optimized manufacturing and processing parameters [11].

In parallel, advances in digital dentistry have allowed the use of additive manufacturing techniques, including 3D printing, which enable rapid production, reduced material waste, and fabrication of complex geometries [12]. Computer-Aided Design – Computer-Aided Manufacturing (CAD-CAM) technology has become a very important innovation to restorative dentistry, comprising a digital design phase (CAD) followed by either subtractive (milling) or additive (printing) manufacturing phase (CAM) [13]. Additively manufactured restorations provide several advantages including material efficiency, batch production capability, customization of internal structures, and highly individualized fabrication [12, 13]. Both printed and milled materials have demonstrated color stability when adequately polished [14–17].

From a biomechanical perspective, 3D-printed resin-based materials may be suitable for posterior rehabilitations due to their elastic behavior, which more closely approximates that of dentin and may contribute to improved stress distribution under functional loading. Recent in vitro studies have reported that 3D-printed resin-matrix ceramic and resin-based crowns can achieve mechanical performance and marginal accuracy comparable to subtractively manufactured alternatives, while offering advantages in manufacturing reproducibility [13, 17].

However, although certain ceramic-filled and nanohybrid printable resins are indicated by manufacturers for use in permanent restorations, their indication as definitive prostheses remains controversial. Several studies have reported limitations related to wear resistance and long-term mechanical stability under functional loading, particularly in posterior regions, leading to frequent recommendations for their use as long-term provisional or semi-permanent restorations. While recent material developments aim to improve their mechanical performance, long-term clinical evidence supporting their use as definitive posterior restorations remains limited [13, 16, 18].

Accordingly, clinical evidence regarding the performance of additively manufactured (3D-printed) resin-matrix ceramic crowns in posterior regions remains scarce. Several factors may hinder their widespread clinical use in load-bearing areas, including concerns related to fracture resistance, wear behavior, long-term fatigue performance under occlusal forces, potential anisotropy related to the layer-by-layer fabrication process, or technique-sensitive post-processing procedures [17–19]. Consequently, further prospective clinical studies are required to better define the indications, limitations, and long-term performance of 3D-printed resin-matrix ceramic crowns in posterior regions [19].

Therefore, the aim of this prospective clinical study was to evaluate the clinical performance and survival rate of posterior printed resin-matrix ceramic crowns fabricated in a fully digital workflow over a two-year observation period. The null hypothesis tested was that no differences would be found between baseline and two-year follow-up for the mechanical and biological parameters assessed. The alternative hypothesis proposed that differences would be detected between baseline and the two-year follow-up for the mechanical and biological parameters assessed.

## Material and methods

### Study population

A total of 62 patients requiring posterior crown were screened and examined from those attending the Master’s Program in Buccofacial Prosthesis and Occlusion (Faculty of Dentistry, Universidad Complutense of Madrid, Spain). Out of these, 30 patients (17 men and 13 women) between 20 and 80 years of age, met the inclusion criteria and were enrolled in the study. Sample size calculation was based on previous investigations [2–4, 9, 14, 15, 20].

Before participation, all patients were informed about the objectives of the study, the clinical procedures, the materials used, the risks and benefits of ceramic restorations, and alternatives to the proposed treatment. All patients signed a written informed consent form for inclusion in this study. The study protocol was approved by the Ethical Committee of Clinical Trial at S Carlos University Clinical Hospital (Madrid, Spain) (C.I. 22/390-EC-P) and conducted in accordance with the Declaration of Helsinki. Treatments were performed between October 20, 2022, and April 10, 2023.

The inclusion criteria comprised: a posterior tooth (molar or premolar) requiring a crown, vital abutments or with adequate endodontic treatment, abutment not previously restored with a crown, periodontal healthy abutments without signs of bone or periapical resorption, sufficient occlusogingival height, stable occlusion, and presence of natural antagonist teeth. The exclusion criteria included: patients with reduced coronal height (< 3 mm), poor oral hygiene, high caries activity, active periodontal disease, and probable bruxism.

### Clinical procedures

A total of 30 posterior crowns (*n* = 30) were fabricated, and each patient has received one crown. The procedures were performed by a single experienced clinician. All patients underwent professional prophylaxis and received oral hygiene instructions prior to treatment.

**Fig. 1:**
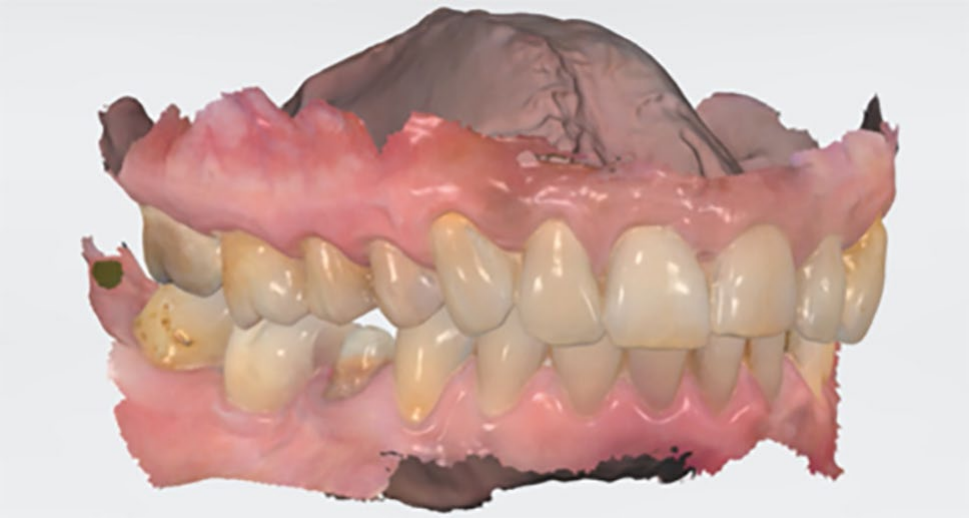
Digital image of a scanned preparations of the mandibular second premolar

A silicone key was obtained prior to preparation for fabrication of temporary crown. Tooth preparation was performed in a standardized way as follows: circumferential 1-mm chamfer, 1-mm axial reduction, 1.5 to 2 mm occlusal reduction, and 10 to 12 degrees axial convergence. A gingival retraction cord was placed before impression. After preparation, full-arch digital impressions were taken using an intraoral scanner (Trios 4, 3Shape, Copenhagen, Denmark) (Fig. 1). Temporary crowns were fabricated using a bis-acrylic composite resin material (Protemp Crown, 3 M ESPE, Seefeld, Germany) and cemented with eugenol-free temporary cement (Telio CS Link, Ivocal Vivadent, Schaan, Liechtenstein). Shade selection was performed using the VITA Classic guide (Vita Zahnfabrik, Bad Säckingen, Germany).

**Fig. 2:**
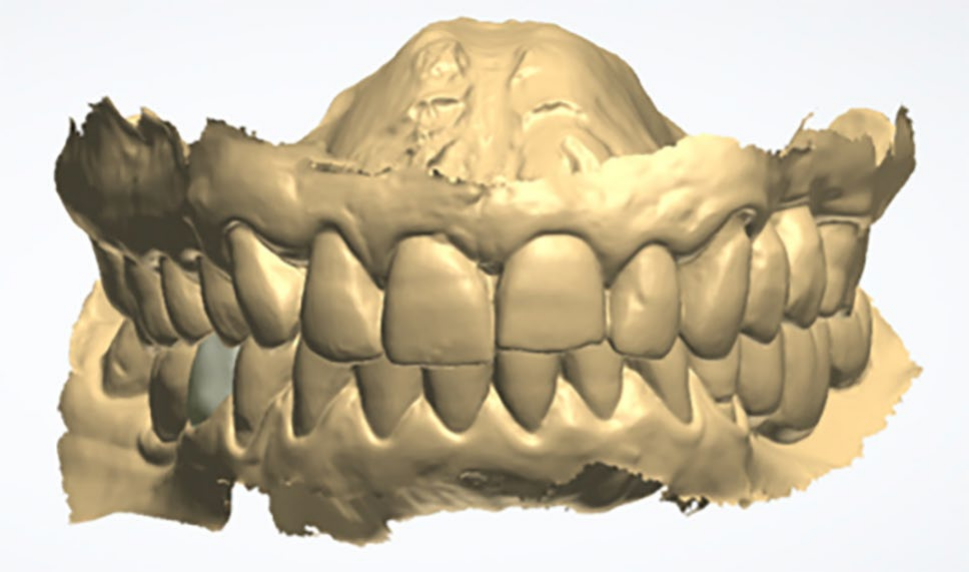
Digital image illustrating the design of a mandibular second premolar crown

**Fig. 3:**
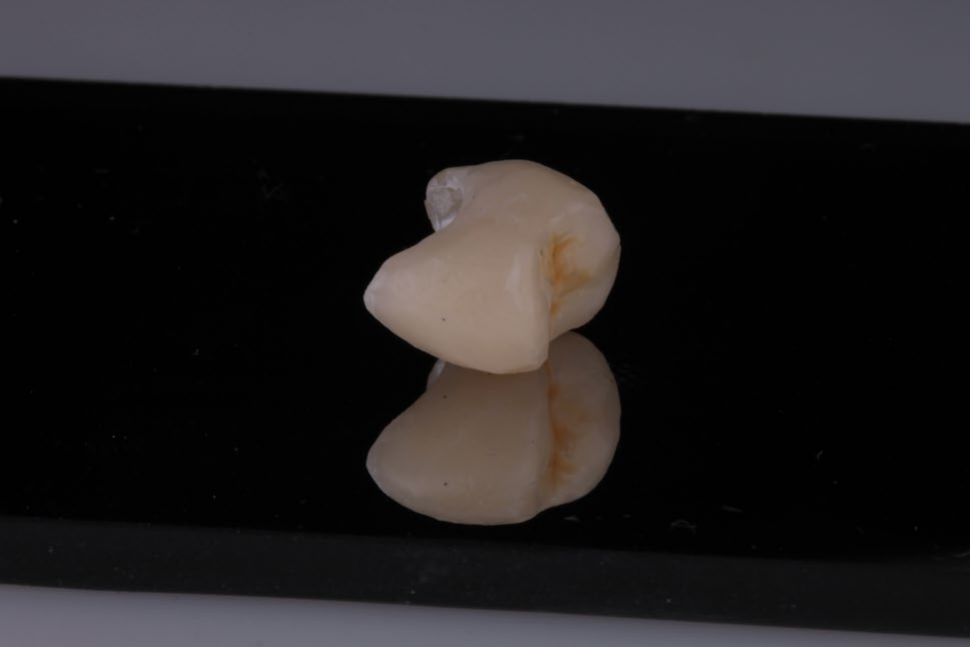
Final printed resin-matrix ceramic restoration

The Standard Tessellation Language (STL) files were imported into the CAD software (DentalCAD, Exocad, Darmstadt, Germany) and exported to the printing software (BEGO CAMcreator Print, Bego, Bremen, Germany) (Fig. 2). The resin-matrix ceramic crowns were fabricated in accordance with the manufacturer´s instructions using a digital light processing (DLP) 3D printer (Varseo XS, Bego). Printing was performed at a temperature of 20–32 ºC with a build orientation of 25º, employing VarseoSmile Crown^plus^ (Bego). This ceramic-filled resin-matrix material is indicated by the manufacturer for definitive single-unit CAD–CAM restorations and was selected because it represents a recently introduced printable material with promising mechanical properties, for which robust prospective clinical evidence, particularly in posterior regions, remains limited [13]. After printing, the crowns were cleaned with ethanol (96%) in an ultrasonic bath for 3 min and cured in a polymerizing unit (Otoflash G171, NK-Optik, Baierbrunn, Germany) with 2 × 1.500 flashes for 3 min [21]. Final finishing and polishing were performed in an external dental laboratory following the manufacturer’s recommendations, using a composite polishing kit (Kit Nº.4652, Komet Brasseler, Lemgo, Germany) (Fig. 3).

**Fig. 4:**
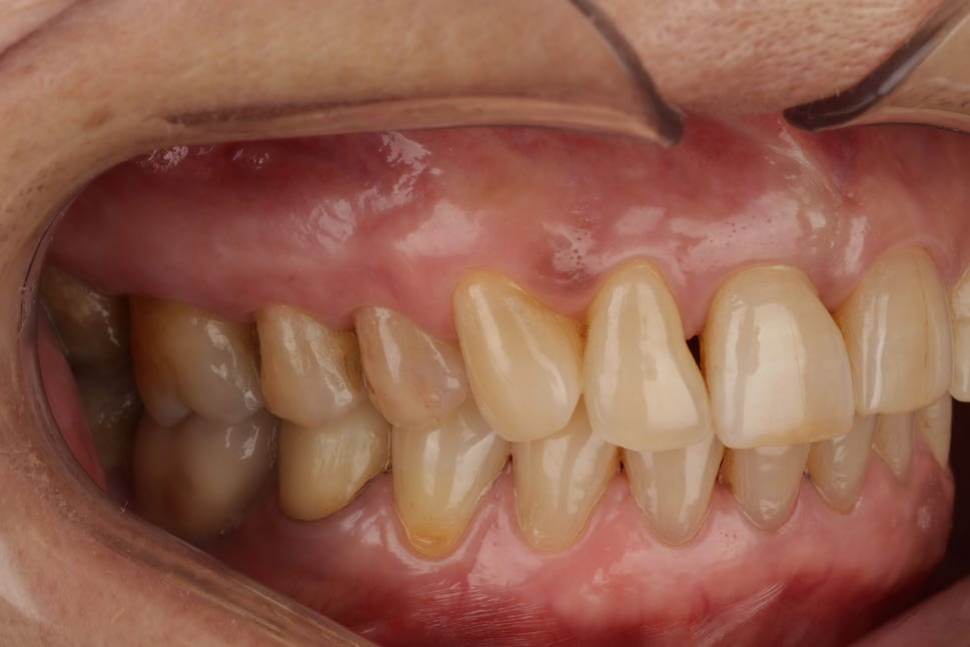
Clinical view of a 3D-printed resin-matrix ceramic crown on a mandibular second premolar after 2 years of function

Before cementation, crowns were tested in the mouth and the contact points, occlusion and marginal fit were evaluated. Then, the crowns were sandblasted with 50 μm aluminum oxide particles at a pressure of 1 bar, ultrasonically cleaned in distilled water for 5 min, and treated with a universal primer (Monobond Plus, Ivoclar Vivadent). Abutment teeth were etched with 37% orthophosphoric acid (15–30 s enamel, 15 s dentin), rinsed and dried. Universal adhesive (Adhese Universal, Ivoclar Vivadent) was then applied to enamel and dentin and light-cured for 10 s. Crowns were cemented using dual-curing resin cement (Variolink Esthetic DC, Ivoclar Vivadent). To facilitate the removal of excess resin cement prior to complete polymerization, an initial tack-curing step of 2 s per surface was performed, followed by definitive light curing for 20 s per surface in accordance with the manufacturer’s recommendations, using a polywave LED curing unit (Bluephase PowerCure, Ivoclar Vivadent; approximately 3000 mW/cm^2^; 385–515 nm) to ensure complete polymerization. Excess cement was carefully removed, and occlusion was evaluated and adjusted to ensure mutually protected occlusion (Fig. 4). The retouched surfaces were carefully polished (Optrafine, Ivoclar Vivadent). All patients were given oral hygiene instructions and scheduled for their check-ups.

### Evaluation

Restorations were assessed using the California Dental Association’s (CDA) Clinical Quality Assessment System for Fixed Prosthesis Restorations [3, 4, 7, 9, 14, 22–25], which includes surface and color, anatomical shape, and marginal integrity. The 30 crowns were examined at baseline (one week after cementation), 6 months, 1 year, and 2 years by two calibrated senior examiners with extensive experience in in the assessment of indirect restorations, who were independent of the restorative treatment and not involved in the clinical procedures. Each researcher evaluated the restoration independently, and disagreements were resolved by assigning the lower score. For periodontal and biological outcome assessment, uncrowned natural teeth located contralateral or antagonistic to the restored abutment teeth were used as control sites. Plaque index (PI), gingival index (GI), probing pocket depth, and margin index (MI) were recorded for both the restored abutment teeth and the corresponding control teeth at each evaluation time point [3, 4, 7, 14, 22, 24, 26–28]. In addition, standardized periapical radiographs using X-ray positioning (Rinn; Dentsply Sirona, Charlotte, NC, USA) and intraoral photographs of the abutment teeth were collected at each recall (Fig. 4).

Success was defined as the crown without any complications during the follow-up period, and survival was defined as the crown that remained in situ at each follow-up visit [6, 29]. Data obtained were entered in Microsoft Excel 16.0 spreadsheet. Descriptive statistics were performed for the following variables: survival and success rate, biological and mechanical complications, CDA ratings, and periodontal parameters. For a better management of the data CDA ratings were numerically coded: excellent = 4, acceptable = 3, repair = 2, replacement = 1. Periodontal parameters were scored on a 0—3 (PI, GI), or 1—4 (MI, PD) scale.

### Statistical analysis

Data analysis was performed with a software (SPSS 22.0, IBM Corp, Armonk, NY, USA). Descriptive statistics were applied for all variables. Wilcoxon’s signed-rank test with Bonferroni correction was applied to paired data to assess differences in CDA scores and periodontal parameters, and for comparisons of periodontal parameters between abutment and control teeth. The Kaplan–Meier Test was applied to assess the probability of survival. Statistical significance was stablished at *α* = 0.05.

## Results

**Fig. 5:**
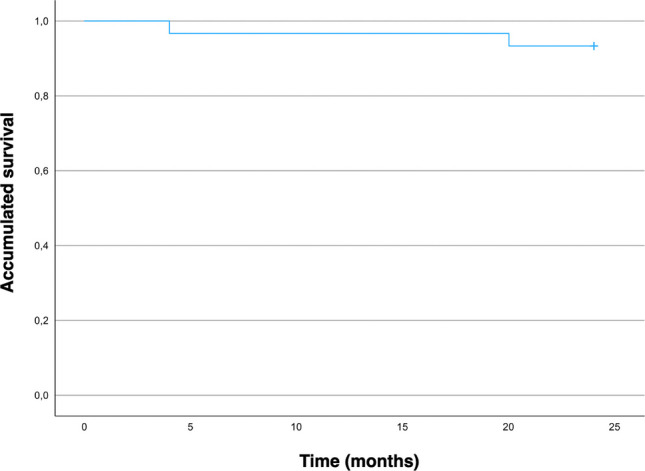
Kaplan Meier cumulative survival rate

**Fig. 6:**
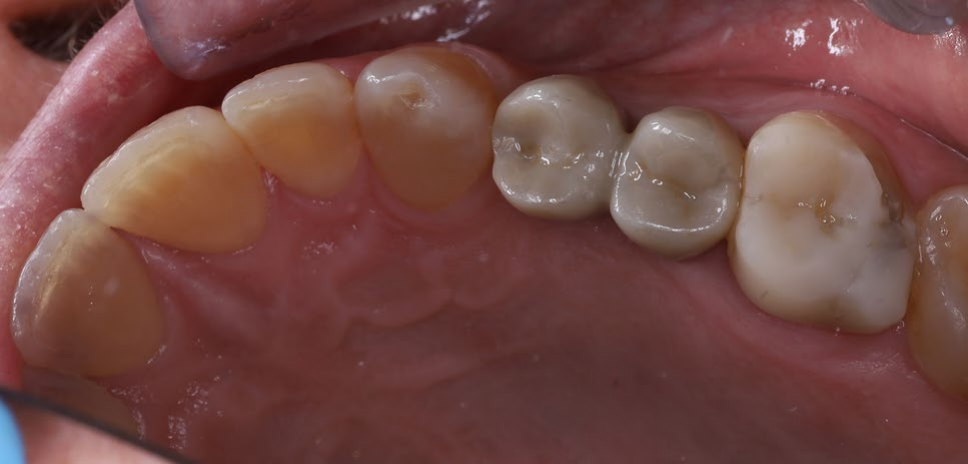
Clinical aspect of a fracture in a maxillary 3D-printed resin-matrix ceramic crown

A total of 30 patients received 30 posterior crowns fabricated from a printed resin-matrix ceramic. The distribution of the restorations is presented in Table 1. No participants were lost during the follow-up period (29 ± 2.6 months). The survival rate was 93% (Fig. 5). Two crowns experienced catastrophic, irreparable failures (Fig. 6). The success rate was 87%, as two additional crowns presented minor cusp fractures that were polished successfully. No biological complications were recorded. One crown debonded at 10 moths and another at 22 months. The crowns were recemented and have remained in function.

**Table 1:** Distribution of restorations

Tooth	Number of restorations
Upper first premolar	4
Upper second premolar	1
Upper first molar	5
Upper second molar	2
Lower first premolar	4
Lower second premolar	4
Lower first molar	4
Lower second molar	6

**Fig. 7:**
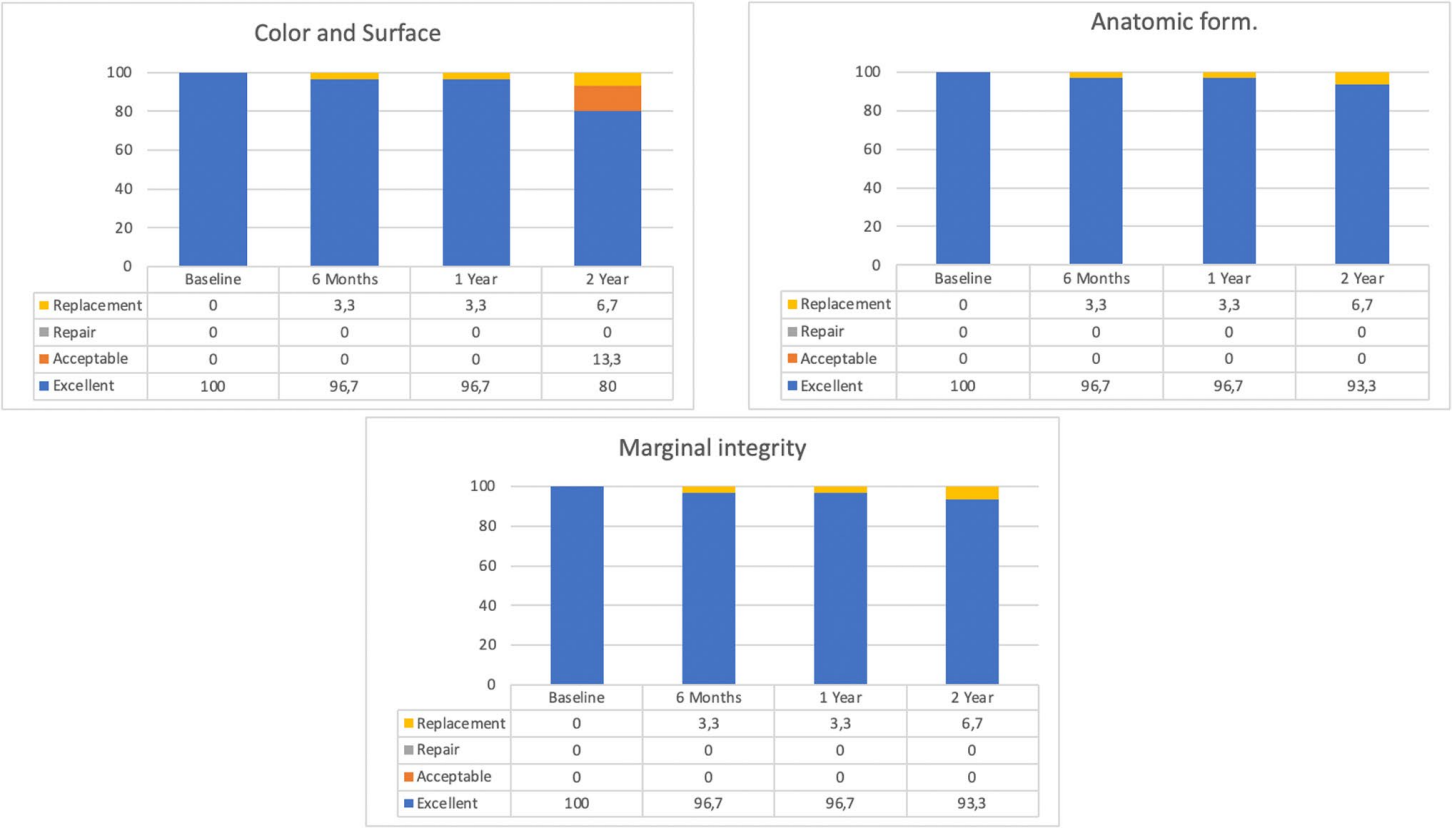
California Dental Association’s criteria rating at baseline, 6 months, 1 year, 2 years and 3 years. Color and surface, Anatomic form and Marginal integrity

At the 2-year evaluation, all crowns were rated within the satisfactory range. Deviations from an excellent rating are shown in Fig. 7. The margin index remained stable throughout the observation period. Differences were observed for color (*p* = 0.046) between baseline to the 2-year follow-up evaluation, due to slight yellow shift in 4 crowns. No other CDA parameters showed differences compared with baseline. Regarding periodontal parameters, the only significant change was an increase in the plaque index at the 2-year evaluation in abutment teeth (*p* = 0.025). No differences were found between abutment and control teeth. No cases of food impaction were reported.

## Discussion

This prospective clinical study evaluated 30 posterior crowns fabricated from a printed resin-matrix ceramic over a 2-year follow-up. Survival, success, complications, and clinical performance were assessed. The results support the rejection of the null hypothesis, as 2 crowns fractured mechanically, and significant differences were observed in color and plaque index values between baseline and the 2-year evaluation.

Metal-ceramic crowns remain the reference standard due to their favorable mechanical properties, longevity, and acceptable aesthetics [29]. Nonetheless, increasing esthetic demands in recent decades have promoted the development and widespread use of ceramic restorations for both anterior and posterior teeth [11, 14, 30]. Hybrid ceramic materials reinforced with a resin matrix have recently gained interest owing to their dentin-like elastic modulus and simplified repair using composite resin [11, 30]. These materials consist of an organic matrix filled with ceramic particles [11]. In the study, a nanoceramic resin containing an organic matrix reinforced with nanoceramic fillers was used.

Parallel to advances in materials, CAD-CAM technology has evolved considerably. Subtractive manufacturing remains the most widely used method for fixed restorations, but it has inherent limitations such as potential crack initiation during milling, tool wear, material waste, and challenges in reproducing complex geometries [31, 32]. Additive manufacturing, by contrast, is gaining importance in dentistry and offers advantages including reduced material waste and enhanced geometric accuracy [12, 13, 17, 33–36]. However, despite its widespread use for provisional restorations, evidence supporting its use for definitive prostheses remains limited [17, 19]. Different additive manufacturing technologies, including stereolithography (SLA), digital light processing (DLP), and liquid crystal display (LCD), may influence the mechanical behavior and long-term performance of printed restorations. In the present study, DLP technology was used, which allows simultaneous layer polymerization and may provide more homogeneous curing; however, differences in interlayer bonding and anisotropy inherent to additive manufacturing may still affect long-term clinical outcomes [17, 19, 37, 38].

To the authors´ knowledge, this is the first clinical study evaluating definitive crowns fabricated from printed resin-matrix ceramic. Consequently, direct comparisons with the literature are not feasible, but the present findings can be interpreted in the context of clinical studies on other ceramic materials and in vitro investigations of printed crowns. The survival rate aligns with previous studies for monolithic zirconia [2, 39] and lithium disilicate crowns [40]. No differences in CDA performance criteria were observed at 6 months, 1 year, or 2 years, except for color changes. All crowns were rated satisfactory, consistent with previously reported outcomes for zirconia restorations [3, 4, 14, 22, 30, 40]. Previous in vitro study reported color shifts towards yellow in a resin nanoceramic after aging, consistent with the findings of the present study [41]. Additionally, other authors have reported lower color stability in 3D printed hybrid ceramic crowns compared with milled counterparts [42], and recommended glazing to optimize color stability [41].

Periodontal outcomes remained stable throughout the observation period, with no significant changes in gingivitis index or probing depth and no differences were obtained between abutment and control teeth. These results were consistent with several previous zirconia studies [2, 4, 30, 40], and contradictory to others [9], suggesting favorable response of the soft tissues. However, an increase in plaque index was observed at one year of clinical follow-up in both abutments and control teeth. In patients in whom there was an increase in the plaque index, oral hygiene instructions were reinforced. Margin stability was maintained during the observation period, suggesting satisfactory marginal adaptation, favorable material biocompatibility, or possibly the relatively short follow-up period. No esthetic complications necessitating repair or replacement of any of the crowns were observed.

Several authors have evaluated in vitro the mechanical properties, or the marginal fit of resin-matrix ceramics. These studies have reported adequate flexural strength (110–143 MPa) [13, 43, 44] and fracture resistance (1.181–3.548 N) [45–48] for the same material used in this study, supporting its suitability for long-term use. Studies comparing subtractive and additive manufacturing techniques have shown higher fracture resistance for subtractively produced crowns, likely due to differences in chemical composition and production processes. The lower content of inorganic filler of the nanoceramic resins results in a more fluid material, which is necessary for additive manufacturing and may explain the reduced fracture strength compared with milled ceramics [45, 47].

In addition, Çakmak et al. evaluated the influence of cement type on fracture strength, reporting greater fracture resistance with dual-curing self-adhesive resin cement [47]. A dual-curing cement was also used in the present study. Mao et al. [49] assessed the adhesion strength of three hybrid ceramic CAD-CAM materials for definitive crowns: Varseo Smile Crownplus (additive), and Vita Enamic and Grandio Blocs (subtractive) subjected to several surface pretreatments. Hydrofluoric acid etching was not recommended, whereas sandblasting plus the application of silane provided optimal adhesion for both milled and printed materials. In the present study, sandblasting with alumina particles and silane application were performed before cementation. However, two crowns debonded during follow-up, suggesting that the surface pretreatment may have been inadequate.

Another aspect evaluated in vitro was the build angle and its influence on the accuracy of additively manufactured resin-ceramic crowns. In the study a build angle of 25º was selected based on previous studies reporting that angles between 20º and 45º provide higher trueness that 0º or 90º orientations for printed crowns [13, 44, 45]. Surface roughness has also been investigated in several hybrid ceramics, including the material used in this study, wit reported values above the clinical threshold of 0.2 μm [13, 44, 45]. Therefore, finishing and polishing procedures are recommended to obtain a smoother surface to prevent plaque accumulation [13, 38]. The observed increase in plaque index at 2 years in both abutment and control teeth, suggests that this finding may not be directly related to the restorative material.

Abdulkareem et al. evaluated marginal adaptation in CAD/CAM crowns fabricated from milled Vita Enamic, milled Cerasmart, and printed Varseo Smile Crown, reporting the smallest marginal discrepancies for the printed crowns (15 μm) [46]. Similar results have been reported by other authors evaluating the marginal adaptation of milled and printed CAD-CAM crowns [48, 50].

The limitations of the present study include the short follow-up period, absence of a control group, exclusion of bruxers, and the heterogeneous distribution of tooth types and arch positions. Maxillary and mandibular premolars and molars were analyzed together despite being subjected to different biomechanical and occlusal loading conditions, which may limit the generalizability of the results. Longer follow-up periods and larger samples are needed to adequately assess medium- and long-term performance of printed resin–matrix ceramic crowns.

## Conclusions

Within the limitations of this clinical study, the results suggest that crowns fabricated from a resin-matrix ceramic using additive technology within a fully digital workflow exhibit promising short-term outcomes for posterior restorations. After 2 years, the survival rate was 93%. However, long-term and larger-scale clinical trials are required to validate the present results.

## Data Availability

No datasets were generated or analysed during the current study.
